# Which patients with palliative malignant biliary obstruction will benefit most from biliary drainage: Development and validation of a prognostic score

**DOI:** 10.1055/a-2760-6318

**Published:** 2026-01-21

**Authors:** Raphael Olivier, Estelle Antoine, Marie Morvan, Augustin D'Aubigny, Jean Baptiste Nousbaum, Noemie Reboux, Enrique Perez Cuadrado Robles, Lucille Queneherve

**Affiliations:** 1 36655 Hepato-Gastroenterology, CHU Poitiers, Poitiers, France; 2 26990 Hepato-Gastroenterology, CHRU de Brest, Brest, France; 3 55151 Hepato-Gastroenterologie, Centre Hospitalier de Cornouaille, Quimper, France; 4 55647 Hôpital Européen Georges Pompidou, Paris, France

**Keywords:** Pancreatobiliary (ERCP/PTCD), ERC topics, MRCP topics, PTCD/PTCS

## Abstract

**Background and study aims:**

Biliary drainage is performed in palliative malignant biliary obstruction (MBO) to improve patient quality of life and enable chemotherapy. This study aimed to create and validate a prognostic score after biliary drainage in patients with palliative MBO.

**Patients and methods:**

Patients undergoing endoscopic or percutaneous drainage for palliative MBO were included in a multicenter, retrospective study. Probability factors associated with 30-day mortality were evaluated by univariable and multivariable logistic regression in the derivation cohort and a prognostic score was built and evaluated in an independent validation cohort.

**Results:**

The derivation cohort included 262 patients, 55% male, 61% of whom had pancreatic adenocarcinoma, mean age 72 years. Probability factors associated with 30-day mortality identified in the derivation cohort were World Health Organization performance status of 3–4 (odds ratio [OR] 7.7 [2.57–25.0]; +3 points), liver metastases (OR 2.7 [1.06–6.98]; +1 point), other metastases (OR 3.85 [1.57–9.97]; +2 points), leukocytes >12G/l (OR 2.4 [0.94–6.08]; +1 point), total bilirubin > 10.8 mg/dL (OR 4.3 [1.45–15.20]; +2 points) and creatininemia > 0.966 mg/dL (OR 7.3 [2.89–19.86]; +3 points). The multivariable model showed good discrimination, with an area under the receiver operating curve (AUROC) of 0.86 (95% confidence interval 0.80–0.93). The prognostic score was used to define two groups of patients, with a low (0–4 points) or high-probability (> 4 points) of 30-day mortality (3% and 32%, respectively). The AUROC in the validation cohort (192 patients) was 0.72, with 30-day mortality of 7% in the low- probability group and 22% in the high- probability group ( *P* = 0.02).

**Conclusions:**

This score could be used in routine clinical practice to identify patients who have better survival outcomes after biliary drainage in palliative MBO.

## Introduction

Malignant biliary obstruction (MBO) is caused by various diseases, among which pancreatic adenocarcinoma and cholangiocarcinoma are the leading causes, followed by liver metastases. The prognosis for these often unresectable tumors remains poor, with low survival rates; 1 2 in such cases, only palliative chemotherapy and best supportive care can be proposed. Because pronounced cholestasis contraindicates most chemotherapy regimens, biliary drainage should be performed when the patient is deemed fit enough for palliative chemotherapy. In patients receiving best supportive care alone, biliary drainage is also performed to treat acute cholangitis, reduce intense pruritus, and limit consequences of prolonged biliary obstruction. 3 4 5

The different strategies of biliary drainage and the types of stents to place are still a matter of debate, particularly in proximal malignant strictures and modified anatomy. 6 Endoscopic drainage with endoscopic retrograde cholangiopancreatography (ERCP) is currently the first choice for unresectable malignant distal biliary obstruction (MDBO) for patients who have normal anatomy. The European Society of Gastrointestinal Endoscopy recommends palliative drainage of malignant hilar strictures by means of ERCP, percutaneous transhepatic biliary drainage (PTBD), or a combination of the two, depending on local expertise. 7 However, the percutaneous approach also can be proposed as the first line in patients with modified anatomy or when ERCP is not available. 7 Both techniques have a non-negligible rate of adverse events (AEs), particularity post-ERCP pancreatitis 8 9 and percutaneous-related complications such as pneumothorax. 10 11 Finally, PTBD and ERCP techniques are not competitors and can be combined in “rendezvous” techniques or complex drainages, particularly in cases of proximal MBO. All these techniques are generally performed on patients under sedation or general anesthesia, both of which carry their own morbidity.

Considering the morbidity and the mortality associated with these techniques, it remains important to assess the risk-benefit profile of the procedure. This means assessing patient life expectancy, the overall therapeutic project, and whether their general condition and comorbidities make them eligible for these procedures, which carry considerable risks. Thus, given all these therapeutic options, the decision-making strategy should involve a multidisciplinary team and be based on patient characteristics and institutional experience. Some prognostic scores have been developed to identify patients at high probability of severe morbidity or mortality of PTBD or biliary stenting, but these do not include ERCP techniques. 12 13 To our knowledge, there is no prognostic score to determine overall survival (OS) after drainage for palliative MBO that would help physicians plan the palliative project, whatever the cause of MBO and type of drainage technique used.

The aim of this study was to create and validate a prognostic score using factors associated with 30-day mortality to characterize the best candidates for palliative biliary drainage in cases of MBO and avoid futile drainage interventions.

## Patients and methods

### Derivation and validation cohorts

In this observational retrospective study, all consecutive adult patients with palliative MBO (unresectable tumor due to the extent of the cancer and/or patient comorbidities), treated with palliative chemotherapy or best supportive care alone and who had undergone biliary drainage (ERCP or PTBD), were eligible. Patients were identified through in-hospital coding and with the medical information department. Inclusion criteria included biological cholestasis, defined as total bilirubin > 2.34 mg/dL and alkaline phosphatase > 1.5 times upper limit of normal, and presence of bile duct dilatation upstream of a tumor lesion visible on imaging and/or with pathology-proven tumor cells. We first created the prognostic score based on a retrospective derivation cohort from one tertiary center. Patients enrolled in the group to establish the derivation cohort score were included between April 2014 and August 2018 and were followed for at least 2 years to evaluate OS. Once the score was established, it was validated in a validation cohort of patients from a second tertiary center, Poitiers University Hospital, using the same inclusion criteria and over the same period to avoid bias related to use of new techniques and endoscopy equipment in more recent years. There was no patient and public involvement during the design, conduct, reporting, interpretation, or dissemination of the study.

### Data collection

The following clinico-biological parameters known to influence prognosis after biliary drainage 14 15 16 were collected: age, sex, body mass index (BMI), percentage of weight loss in the previous 3 months, American Society of Anesthesiologists (ASA) score, World Health Organization performance status (WHO PS), comorbidities, sepsis, pruritus, presence of liver metastases, type of cancer, and location of biliary obstruction. Comorbidities collected were diabetes mellitus, heart disease (including valvular, hypertensive and coronary heart disease), chronic lung disease (including chronic obstructive pulmonary disease and chronic pulmonary fibrosis), chronic renal failure, stroke, and cirrhosis. The following biological data were collected in the 48 hours preceding the biliary procedure: hemoglobin, platelets, leukocytes, albumin, total bilirubin, and prothrombin time. Patient follow-up data were also recorded: date of death or date of last follow-up, length of hospital stay (i.e. time between the first biliary drainage and patient discharge), presence/absence of elevated bilirubin at 30 days (bilirubin cut-off at 2.34 mg/dL), and treatment with chemotherapy or not. Drainage-related AEs, including acute pancreatitis, sepsis, perforation, and bleeding, were collected. The Adverse Events Gastrointestinal Endoscopy (AGREE) classification was used for both ERCP and PTBD to analyze drainage morbidity. 17 18

Missing data, inherent to the retrospective nature of the study, are detailed in Table 1 .

### ERCP and PTBD procedures

ERCP was performed as routine clinical practice in the center, often as first-line treatment of MBO and under general anesthesia. Sphincterotomy was left to physician discretion. The type of stent used was collected: plastic stent, self-expandable metal stent (SEMS) or fully-covered self-expandable metal stent. A patient could undergo several interventions in the event of stent obstruction.

PTBD was performed under general anesthesia in cases of SEMS placement. Local anesthesia could be used in cases of external biliary drainage.

Interventions were performed in expert centers, according to procedures recommended by international guidelines. All operators were experienced, having performed over 500 procedures. For both procedures, technical success was defined as successful placement of the biliary stent and reduction in total bilirubin levels to below twice the upper limit of normal. In cases of malignant proximal biliary obstruction (MPBO), choice of ERCP or PTBD was left to the discretion of the physician and the expertise of the center, i.e. interventional radiology or endoscopy teams. If different drainage techniques were used, AE were collected even in the case of failure.

### Statistical analysis

Qualitative data were described as numbers and frequencies and quantitative data were described as means and standard deviations. Categorical variables were compared using a Chi² test or a Fisher exact test. Quantitative variables were compared using a Student's *t* -test.

OS was estimated using the Kaplan Meier analysis and differences between the survival curves (according to the prognostic group) were evaluated using the log-rank test. We analyzed 30-day mortality because this cut-off is a commonly accepted criterion and clinically relevant, and because it would be unreasonable to propose biliary drainage to patients with a life expectancy of less than 30 days.

Univariable analysis of 30-day mortality was then performed using pre-selected variables (age, BMI, pruritus, WHO PS, obesity, ASA score, peritoneal carcinomatosis, liver metastasis, history of diabetes, heart disease, chronic lung disease, chronic renal failure, stroke, cirrhosis, hemoglobin, platelets, leukocytes, total and conjugated bilirubin, urea, and creatininemia) using a simple logistic model for quantitative variable and a Fisher test for qualitative variables. It was considered that in 30 days, chemotherapy had no impact on survival, because the majority of patients waited 3 to 4 weeks for bilirubin to decrease before receiving chemotherapy, which thus had no impact on 30-day mortality. Variables with *P* < 0.2 were retained for multivariable analysis. Thresholds for quantitative variables were obtained by calculating the area under the curve that best described 30-day mortality according to the Youden method. A multivariable analysis was then performed by constructing a multivariable logistic model with the variables retained previously. The model was validated internally using the resampling validation method for logistic models with 250 bootstrap re-samples, and the optimism-corrected receiver operating characteristic (ROC) and discrimination slope (difference in mean of predictions between patients with or without 30-day mortality) with their 95% confidence intervals (CIs) were estimated. In addition, leave-one-out cross-validation was performed to calculate the accuracy of the model (measured as the percentage of correctly classified patients).

A prognostic index of 30-day mortality was developed by assigning a weight to each predictor based on the odds ratios (ORs) from the multivariable final model. These ORs were divided by the smallest OR of the model and rounded to the nearest integer. The score enabled classification of patients into two categories according to probability of mortality after the intervention, in accordance with the Youden method, with a threshold of 4. The two groups based on this prognostic index were compared in terms of 30-day mortality (primary endpoint), 90-day mortality, and OS (secondary endpoints). Accuracy of the prognostic index was assessed in the development and validation cohorts.

*P* < 0.05 was considered significant. Data were analyzed using software R version 3.5.1 R core team (Vienna, Austria).

## Results

### Derivation cohort

**Fig. 1:**
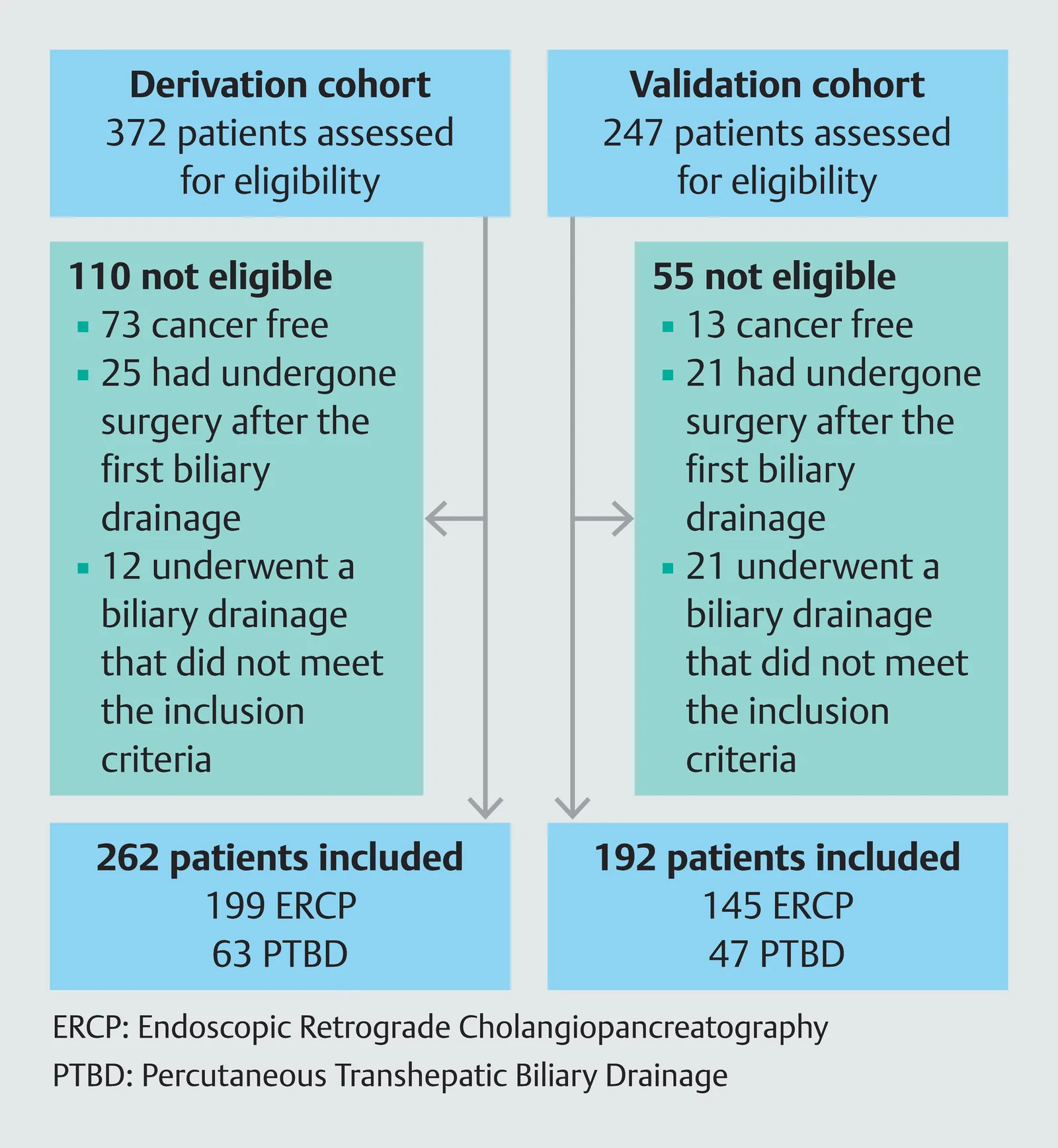
Flow chart.

Of 372 patients screened in the derivation cohort, 262 (55.0% male; median age 73; interquartile range [IQR] 64–82) were included in the analysis ( Fig. 1 ), including 199 and 63 patients in the ERCP and PTBD groups, respectively. The main characteristics of patients are listed in Table 1 . Altogether 373 procedures were performed, 244 ERCPs and 129 PTBDs, with an average of 1.23 procedures per patient. Of the 244 ERCPs, 116 used uncovered stents (47.5%), 64 used covered stents (26.2%), 18 used plastic stents (7.4%), and 46 procedures failed (18.9%). Of the 129 PTBDs, 100 used SEMS (78.7%), 27 used external or internalized biliary drains (21.3%), and there were no procedure failures. All-cause 30-day mortality after the first biliary drainage for MBO in the derivation cohort was 15%. Mortality directly related to the procedure was 3.0%, four of 63 (6.3%) in the PTBD group and four of 199 (2.0%) in the ERCP group.

**Table 1:** Characteristics of patients presenting with malignant biliary obstruction in the two cohorts.

Center	Derivation cohort N = 262 ^1^	Validation cohort N = 192 ^*^
**Sex**		
Male	145 (55.3%)	106 (55.2%)
**Age**	72 (12)	72 (11)
**BMI**	24 (5)	23.6 (4.7)
**WHO PS**		
0–1	137 (52.2%)	123 (64.0%)
2	88 (33.5%)	52 (27.0%)
3–4	37 (14.1%)	11 (5.7%)
**Pruritus**	83 (31.9%)	87 (45.3%)
**Sepsis**	49 (18.7%)	27 (14.0%)
**Heart disease**	71 (27.0%)	27 (14.0%)
**Chronic lung disease**	25 (9.5%)	20 (10.4%)
**Chronic renal failure**	18 (6.8%)	3 (1.5%)
**Obesity**	27 (10.3%)	22 (11.4%)
**Diabetes**	47 (17.9%)	48 (25.0%)
**Stroke**	16 (6.1%)	9 (4.7%)
**Cirrhosis**	16 (6.1%)	3 (1.6%)
**Peritoneal carcinomatosis**	36 (13.7%)	36 (18.7%)
**Liver metastases**	93 (35.4%)	76 (39.5%)
**^†^ Hemoglobin g/dl **	11.3 (1.6)	11.5 (1.8)
**^†^ Platelets /mm3 **	296 (122)	299 (112)
**^†^ Leukocytes G/l **	9.7 (5.8)	9.5 (4.5)
**^†^ Total bilirubin mg/dL **	14.0 (8.5)	16.9 (9.4)
**^†^ Conjugated bilirubin mg/dL **	11.9(7.7)	15.9 (8.9)
**^†^ Creatininemia mg/dL **	0.784 (0.466)	0.977 (0.784)
**^†^ Albuminemia g/L **	29 (6)	32 (6)
**Obstruction causes**		
Primary tumor	203 (77.5%)	160 (83.3%)
Metastatic lesion	59 (22.5%)	32 (16.6%)
**Obstruction site**		
MDBO	184 (70.2%)	122 (63.5%)
MPBO	78 (29.8%)	67 (40.7%)
Main bile duct	184 (70.2%)	112 (59.3%)
Common hepatic duct	24 (9.2%)	22 (11.4%)
Hilar	24 (9.2%)	30 (15.6%)
Intra hepatic bile duct	30 (11.4%)	15 (7.8%)
Missing data	0	3
**Cancer type**		
Pancreatic adenocarcinoma	153 (60.7%)	119(61.9%)
Cholangiocarcinoma	53 (20.2%)	52(27.0%)
Colorectal cancer	20 (9.4%)	11 (5.7%)
Ampullary cancer	16 (7.5%)	5 (2.6%)
Lung cancer metastasis	10 (4.7%)	0
Gastric cancer metastasis	9 (4.2%)	0

^*^ Headcount (%) or average (standard deviation).

^†^ Biological tests performed during hospitalization on the day of drainage.

MDBO, malignant distal biliary obstruction; WHO PS, World Health Organization performance status.

### Factors associated with 30-day mortality

**Fig. 2:**
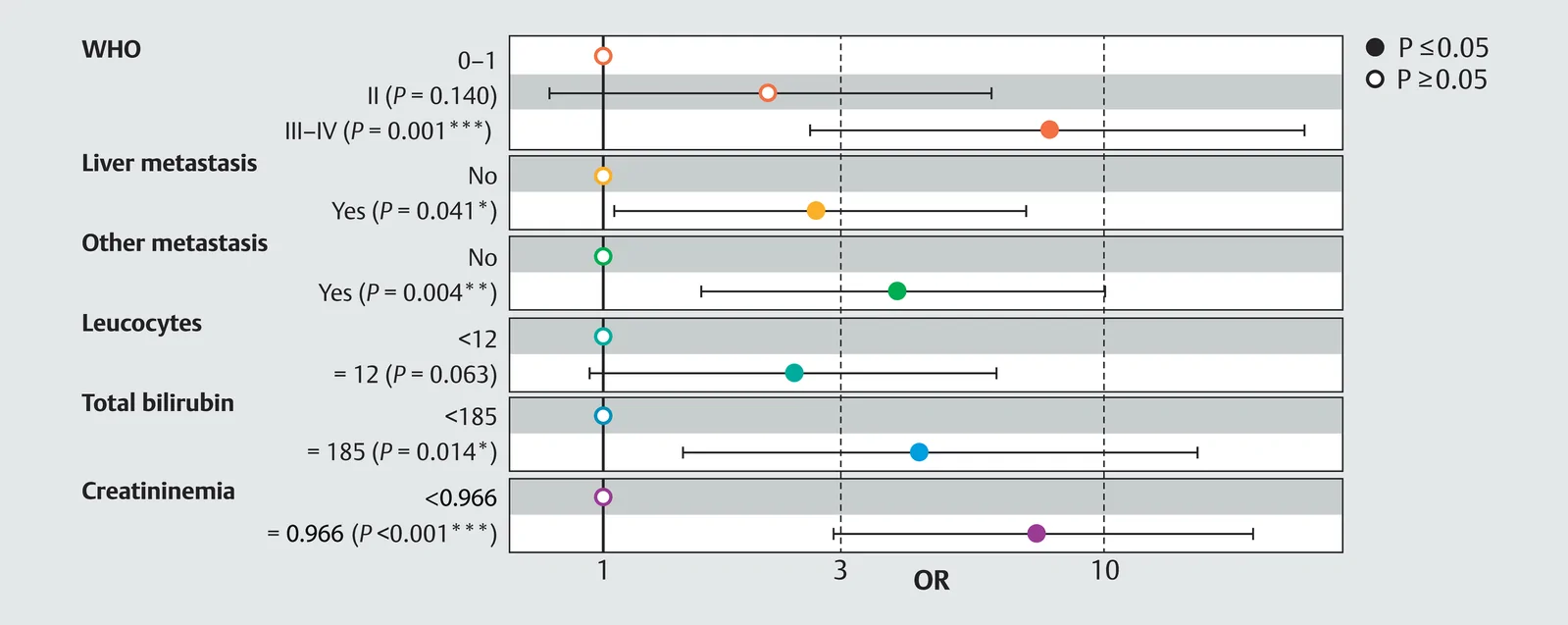
Predictive factors for 30-day mortality in multivariable analysis. OR, odds ratio; WHO, World Health Organization performance status.

Table 2 shows univariable and multivariable analyses of factors independently predictive of 30-day mortality. All the analyzed factors are presented in **Supplementary Table 1** . The factors found to be predictive in the final model ( Fig. 2 ) were a WHO PS of 3 or 4 (OR 7.7; 95% CI 2.57–25.0; *P* < 0.001), liver metastases (OR 2.7; 95% CI 1.06–6.98; *P* = 0.041), other metastases (non-liver metastases and non-peritoneal carcinomatosis) (OR 3.85; 95% CI 1.57–9.97; *P* = 0.004), leukocytes > 12 G/l (OR 2.4; 95% CI 0.94–6.08; *P* = 0.063), total bilirubin > 10.8 mg/dL (OR 4.3; 95% CI 1.45–15.20; *P* = 0.014) and creatininemia > 0.966 mg/dL (OR 7.3; 95% CI 2.89–19.86; *P* < 0.001).

**Table 2:** Univariable and multivariable analyses of factors predictive of 30-day mortality.

		Univariable analysis		Multivariable analysis	
Characteristic	N	OR (95% CI)	*P* value	OR (95% CI)	*P* value
**Leukocytes ≥ 12 G/L**	**260**	**1.5 (0.76–2.24)**	**< 0.0001**	**0.97 (-0.01–1.94)**	**0.05**
**Total bilirubin ≥ 10.8 mg/dL**	**258**	**1.37 (0.47–2.48)**	**0.006**	**1.43 (0.33 to -2.72)**	**0.02**
**Creatininemia ≥ 0.966 mg/dl**	**261**	**1.79 (1.05–2.54)**	**< 0.0001**	**1.54 (0.38 to -2.73)**	**0.009**
**WHO PS**	**262**				
0–1		Reference			
2		0.72 (-0.13–1.6)	0.1	0.62 (0.40–1.67)	0.2
3–4		1.93 (1.03–2.85)	< 0.0001	1.84 (0.72 to -3.09)	0.003
**Liver metastases**	262	0.94 (0.24–1.65)	0.008	0.91 (-0.08–1.93)	0.07
**Other metastases**	262	1.34 (0.63–2.09)	0.0003	1.20 (0.2 to -2.23)	0.02

CI, confidence interval; OR, odds ratio; WHO PS, World Health Organization performance status.

### Model development and performance

**Fig. 3:**
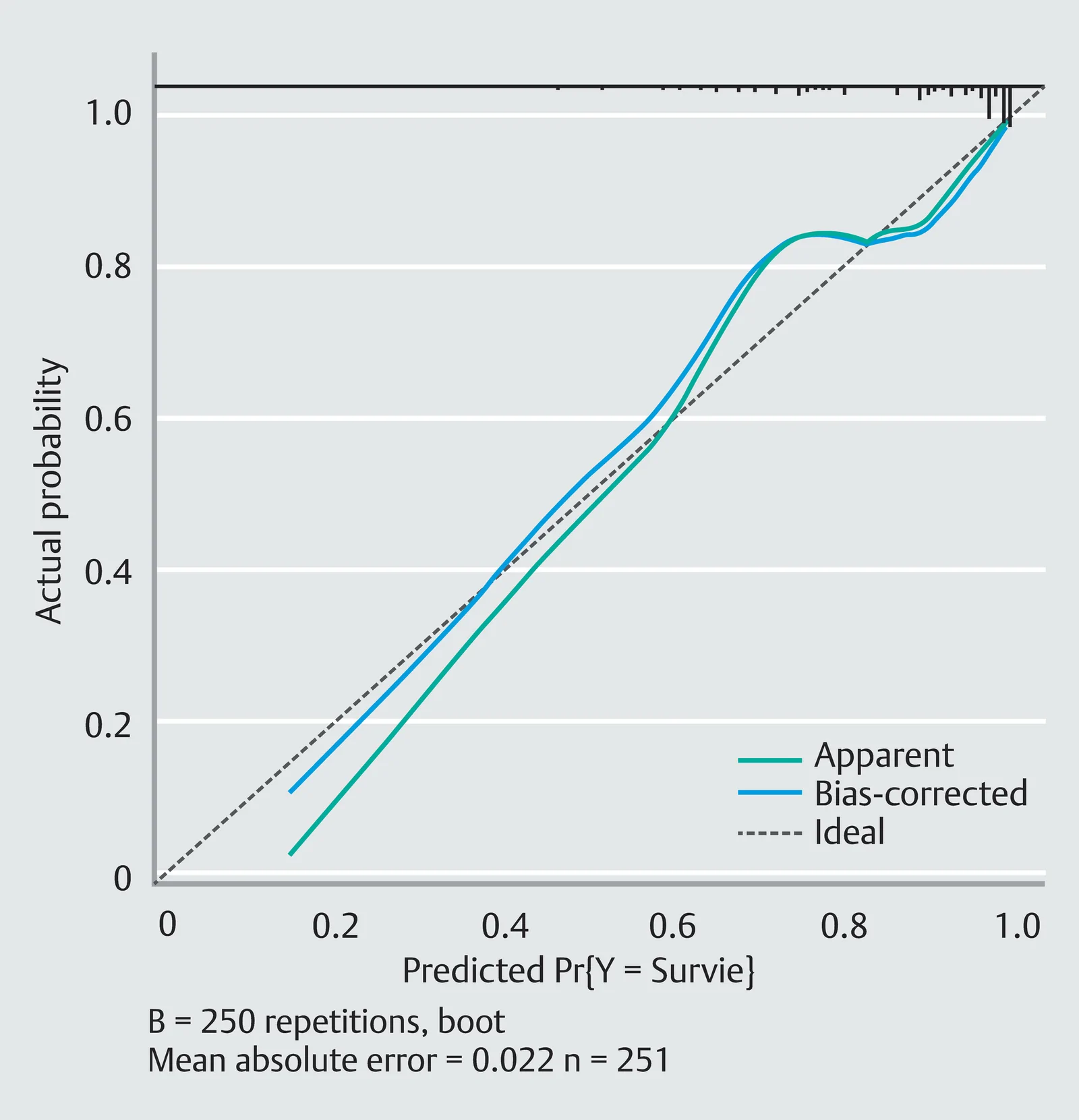
Calibration curve for multivariable regression.

The multivariable model had an area under the ROC curve of 0.86 (95% CI 0.80–0.93), showing good discrimination ( **Supplementary Fig. 1** ). Calibration of the model was fair according to the calibration curves ( Fig. 3 ) and to the Hosmer–Lemeshow test results (X2 = 7.56; *P* = 0.48). The discrimination slope was 0.033. In addition, accuracy of the model was measured at 88.0%.

ORs for the final model were used to develop the prognostic score as shown in Table 3 , taking values between 0 and 11. In the group with a low probability of 30-day mortality (score ≤ 4), 30-day mortality was 3.0%, compared with 32.0% in the group with a high probability of 30-day mortality (score > 4).

**Table 3:** Final 30-day mortality score.

Prognostic factor	Final model		Score Points
	OR (95% CI)	*P* value	
**WHO PS 2**	-0.757 (-1.78–0.24)	0.139	**+1**
**WHO PS 3–4**	-2.051 (-3.22 to -0.94)	0.0004	**+3**
**Liver metastases**	-0.978 (-1.94 to -0.05)	0.041	**+1**
**Other metastases (non-liver and non-peritoneal metastases)**	-1.349 (-2.3 to -0.45)	0.004	**+2**
**Leukocytes ≥ 12G/L**	-0.88 (-1.8–0.06)	0.063	**+1**

CI, confidence interval; OR, odds ratio; WHO PS, World Health Organization performance status.

Patients were at high-risk of 30-day mortality if score > 4.

### Validation cohort

Overall, 247 patients were screened for the validation cohort, 192 of whom were included (55.0% male, median age 73; IQR 64–81), 145 in the ERCP group and 47 in the PTBD group. Altogether 262 procedures were performed, 142 ERCPs and 127 PTBDs, with an average of 1.36 procedures per patient ( Fig. 1 ). Sixteen ERCP procedures (11.0%) and two PTBDs (1.4%) failed. The main characteristics of patients are listed in Table 1 .

There were significant differences between the derivation and validation cohorts in the WHO PS (52.0% WHO PS 0–1 vs. 66.0%; *P* = 0.003), heart disease history (73.1% vs. 86.2%; *P* < 0.001), mean total conjugated bilirubin level (11.9 mg/dL vs. 15.9 mg/dL; *P* < 0.001), creatininemia (0.784mg/dL vs. 0.977mg/dL; *P* < 0.001), and albuminemia (29 g/L vs. 32 g/L; *P* < 0.001), respectively.

Thirty-day all-cause mortality after the first biliary drainage for MBO in the validation cohort was 17.0%.

When applied to the validation cohort, accuracy of the score was 61.0%, with 13 of 18 patient deaths correctly classified. In the low-probability group (score ≤ 4), 7.0% of patients died within 30 days compared with 22.0% of patients in the high- probability group (score > 4).

Mortality directly related to the procedure was twice as high in this cohort (6.3% versus 3.0%, *P* = 0.012), with mortality notably higher in the PTBD group (13 of 127; 10.2%) than in the ERCP group (4 of 142; 2.8%).

### Ninety-day mortality and overall survival

In the derivation cohort, 90-day mortality was 19.0% (95% CI 13–25) in the low-probability group versus 59.0% (95% CI 49–68) in the high-probability group ( *P* < 0.0001). In the validation cohort, 90-day mortality was 22% (95% CI 12–31) in the low-probability group versus 42.0% (95% CI 28–53) in the high-probability group ( *P* = 0.02).

**Fig. 4:**
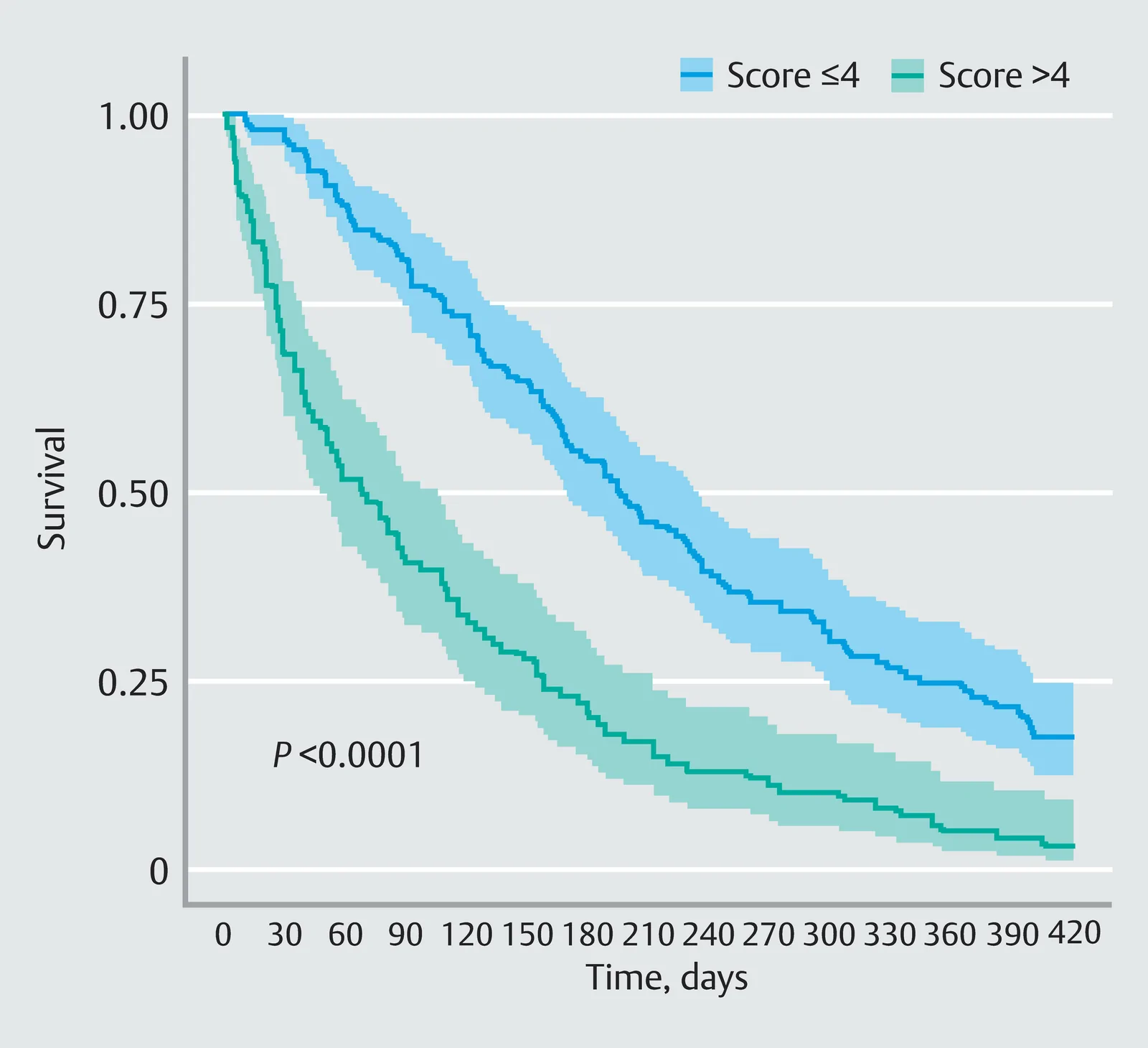
Overall survival according to the final score in the derivation cohort.

**Fig. 5:**
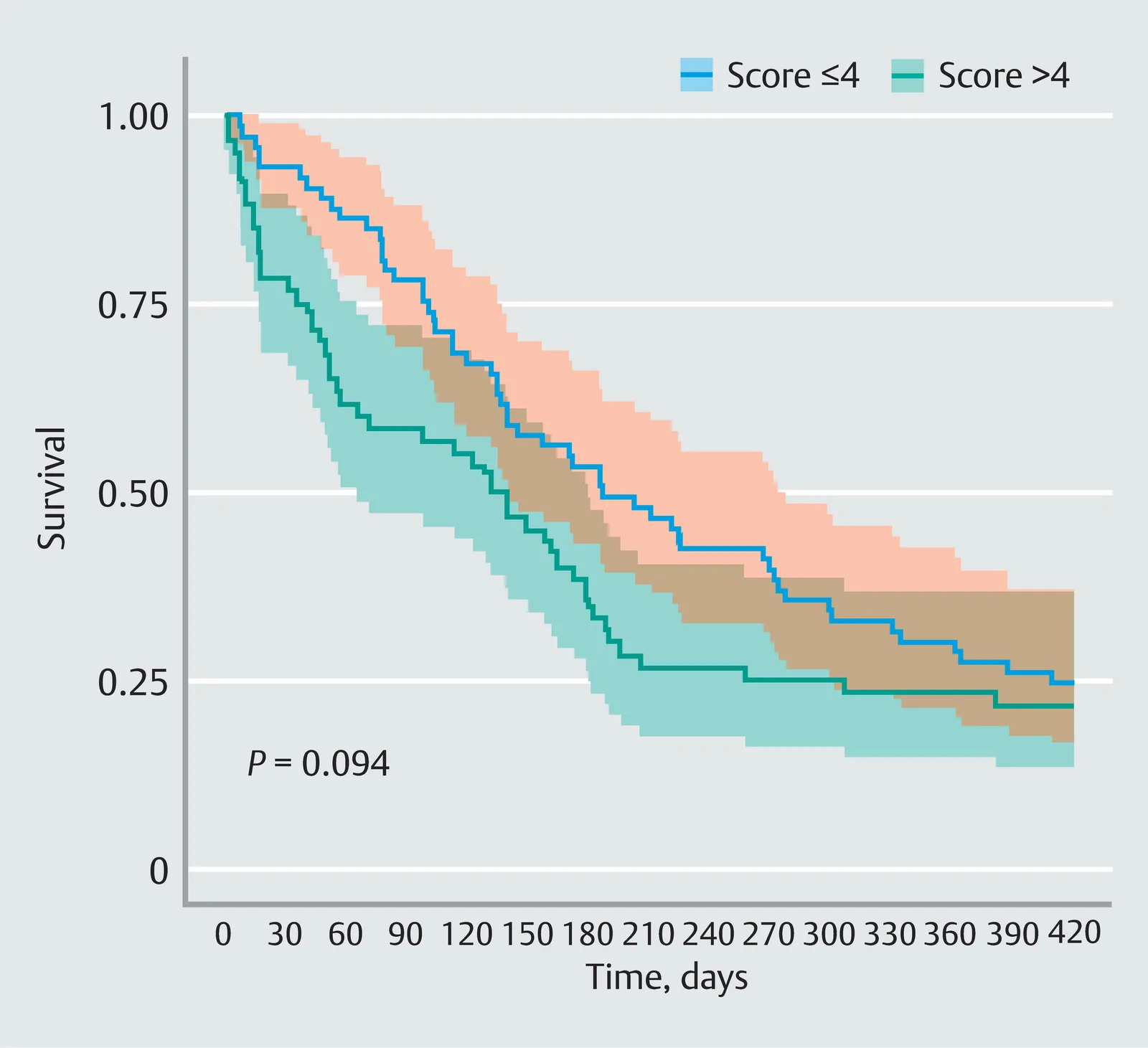
Overall survival according to the final score in the validation cohort.

In the derivation cohort, OS was 274 days in the low-probability group versus 110 days in the high-probability group ( *P* < 0.0001) ( Fig. 4 ). In the validation cohort, it was 298 days in the low-probability group versus 228 days in the high-probability group ( *P* = 0.094) ( Fig. 5 ).

### Biliary drainage morbidity

AEs are shown in Table 4 and Table 5 . In the derivation cohort, the AE rate was 18.2% (68/373). The most frequent AE was sepsis (9.4%, 35 events), followed by hemorrhage (4.5%, 17 events), post-ERCP pancreatitis (3.2%, 12 events), and perforation (1.0%, 4 events).

**Table 4:** Adverse events according to AGREE classification.

AGREE	Derivation cohort (n = 373 interventions)	Validation cohort (n = 269 interventions)
**Grade I**	72 (19.3%)	44 (16.3%)
**Grade II**	61 (16.3%)	31(11.5%)
**Grade IIIa**	6 (1.6%)	4 (1.4%)
**Grade IIIb**	0	0
**Grade IVa**	2 (0.5%)	3 (1.1%)
**Grade IVb**	6 (1.6%)	4 (1.4%)
**Grade V**	8 (2.1%)	17 (6.3%)

**Table 5:** Adverse events in the two cohorts.

	Derivation cohort			Validation cohort		
Adverse event	ERCP (n = 244)	PTBD (n = 129)	Total n = 373)	ERCP (n = 142)	PTBD (n = 127)	Total (n = 269)
Sepsis	15 (6.1%)	20 (15.7%)	35 (9.4%)	17 (11.9%)	23 (19.1%)	40 (15.3%)
Bleeding	14 (5.7%)	3 (2.4%)	17 (4.5%)	4 (2.8%)	5 (4.1%)	9 (3.4%)
Pancreatitis	12 (4.9%)	0 (0%)	12 (3.2%)	1 (0.7%)	2 (1.7%)	3 (1.1%)
Perforation	3 (1.2%)	1 (0.8%)	4 (1%)	0 (0%)	3 (2.5%)	3 (1.1%)
Other	0 (0%)	0 (0%)	0 (0%)	1 (0.7%)	9 (7.5%)	10 (3.8%)
Total	44 (17.9%)	24 (18.9%)	68 (18.3%)	23 (16.1%)	42 (33.0%)	65 (17.5%)

ERCP, endoscopic retrograde cholangiopancreatography; PTBD, percutaneous transhepatic biliary drainage.

A patient could have several procedures.

In the validation cohort, the AE rate was 24.8% (65/262). The most frequent AE was also sepsis (15.3%, 40 events), followed by hemorrhage (3.4%, 9 events), post-ERCP pancreatitis (1.1%, 3 events) and perforation (1.1%, 3 events).

## Discussion

In this retrospective study, several factors associated with 30-day mortality in patients with unresectable MBO treated with endoscopic or percutaneous biliary drainage were highlighted. A score based on seven variables built from a derivation cohort was able to determine mortality with high predictive accuracy; about one-third of patients with a high-probability score died within 30 days following biliary drainage. This score was validated in a second cohort with a predictive accuracy of 61.0%, and, therefore, is promising for evaluating patients with MBO in routine clinical practice.

The majority of previous studies compared only morbidity/mortality related to only ERCP or only PTBD, 19 or different types of endoscopic stents, 20 or radiological drainage. 13 Other scores have been designed to estimate the prognosis of patients with MBO, for example, the prognostic nutritional index and the neutrophil to lymphocyte ratio in all-comer patients, but none in context of biliary drainage. 21 Other, more conventional studies used an inflammation-based prognostic score based on C-reactive protein and albuminemia. 22 23 These scores were built with one or two parameters, in single-center retrospective studies, in fewer than 100 patients, and were not used in routine clinical practice. Some scores were used to guide the choice between the two drainage strategies, ERCP or PTBD, but did not provide an overall prognostic assessment to guide the decision to perform biliary drainage or not. 24 To our knowledge, there is no available score for real-life use that helps physicians decide whether to implement biliary drainage, whatever the technique considered.

Our score, built using a derivation and then a validation cohort, is easy to calculate for use in routine clinical practice and could help physicians manage MBO in numerous situations. It provides an overall prognostic estimate, whatever the type of MBO or drainage used. This score could also be used in non-expert centers, where not all drainage techniques are available, to decide, in consultation with a tertiary center, whether to perform biliary drainage. Users of the score should not intervene in the processing of input data for use with the model, and no specific level of expertise is required.

Cases of MBO are constantly increasing, as are indications for biliary drainage, i.e. cholangiocarcinoma and pancreatic adenocarcinoma. Many patients with MBO are elderly and frail with limited life expectancy, and not necessarily eligible for these risky or even potentially life-threatening procedures. In some cases, if no chemotherapy can be performed, it would seem more reasonable to refrain from carrying out these interventions and encourage a palliative approach with best supportive care alone. Our score provides information on 30-day mortality after drainage for MBO and could help physicians in the decision-making process and warn of the difficulty of carrying out additional chemotherapy, for example, if the procedure itself is high risk. Of course, other parameters must be taken into account. Indeed, in the high-probability group, mortality at 30 days was 32.0% and median OS was 110 days, meaning that physicians cannot decide not to drain according to this argument alone, because some patients have a life expectancy of more than 3 months. However, the score allows physicians to discuss the benefits and risks of biliary drainage with patients and their families and guide the medical strategy in the light of other parameters, such as the possibility of implementing palliative chemotherapy. However, if drainage is considered despite a high-probability score, it will be important to explain to patients and their families the high risk of morbidity and mortality even if biliary drainage is carried out. In contrast, in patients with a low-probability score, the 30-day mortality rate following biliary drainage was 3.0%, which will lead physicians to carry out the procedure to avoid complications of MBO, such as pruritus or sepsis.

We found significant differences between the two cohorts in WHO PS, heart disease history, total bilirubin level, creatininemia, and albuminemia. Patients from the validation cohort seemed to be frailer, which could explain the differences between the two scores and the lower score accuracy of 61.0% in this cohort. In support of this hypothesis, mortality directly related to the procedure was three times higher in the validation cohort. Moreover, a larger proportion of patients had PTBD in this cohort, which may be explained by the fact that in this cohort, it was often performed in frail patients not fit enough to have general anesthesia and/or for more severe/proximal biliary obstruction, which is more difficult to treat. Overall, the AEs were relatively frequent but mainly low grade (1 to 3 according to the AGREE classification). Assessment of 90-day mortality and OS according to the prognostic score in both cohorts provided interesting information about the accuracy of the score over periods longer than 1 month. Significant differences appeared between the groups after application of the score, confirming its validity in the longer term and throughout patient therapeutic course after drainage.

Our study has some limitations. The number of hilar stenoses was rather low, specifically in the derivation cohort (9.2%). Our score, therefore, seems less applicable to this particular situation. One other limitation was use of the AGREE classification for radiological drainage. This classification, although not initially intended for this purpose but for endoscopy procedures, was straightforward and allowed physicians to group the two procedures in the two cohorts, thereby enabling comparisons to be made. It should also be remembered that the teams in the two tertiary centers considered all of the patients in this study to be in good general condition and fit enough to undergo drainage; they would not have proposed the intervention to frailer patients. Given this, it is reasonable to assume that survival in the absence of biliary drainage would have been much lower. One of the aims of drainage is to improve patient quality of life (QoL), by reducing pruritus, for example, as reported in the literature. 25 Nevertheless, when survival is short, less than 30 days, it is uncertain whether biliary drainage improves QoL.

During the last decade, endoscopic ultrasound guided (EUS-guided) biliary drainage using dedicated metallic stents has been a game-changer. 26 27 This relatively new strategy includes EUS-guided choledochoduodenostomy, hepatico-gastrostomy, and gallbladder drainage. Therefore, another limitation of our study was absence of both EUS-guided drainage and lumen-apposing metal stents. This technique was not widely used at the time of the study (2014 to 2018), but has taken off in the years since. Currently recommended in the event of ERCP failure, this technique is now being endorsed by some authors as a first-intention strategy. 28 Some studies and a meta-analysis report similar technical and clinical success rates for drainage by EUS or ERCP, and also no differences in terms of AEs, 29 30 which suggests that EUS can even be considered in frail patients. 31 The technical success rate in the literature ranges from 90% to 100% and the clinical success rate from 85% to 100%. 32 It would be interesting to evaluate our score in patients treated with EUS-guided drainage, but it is reasonable to assume that our score does not depend on the technique used for biliary drainage.

Another limitation is that our two cohorts are heterogeneous, in terms of both lesion types (MDBO and MPBO) and drainage techniques, and these differences could induce obvious analysis biases. Nevertheless, our score remained accurate in the validation cohort, suggesting that it is independent of the type of biliary obstruction and the technique used. In addition, because we intended to develop a “universal” score that was to be used before drainage was performed, the type of drainage procedure was not included in the score. A further limitation is that our score allows a classification between a low and a high probability of 30-day mortality, but not a continuous estimate of survival. However, it is clinically relevant to have a dichotomic classification of patients, and our score is also strongly associated with 90-day mortality and OS of patients.

To our knowledge, this is the first score to predict 30-day mortality in a large series of cases that includes both endoscopic and percutaneous transhepatic drainage in MBO with a validation cohort and robust methodology. We believed that it was important to create a score that would be relevant whatever the procedure used, because in current clinical practice, patients may be eligible for both techniques, and not every medical facility can systematically offer both approaches. This combined score, therefore, is suitable for larger numbers of patients and even in smaller medical centers, which may have only one of the two techniques available. It also will be of interest for non-expert centers that do not have access to biliary drainage because it will help them decide whether to refer patients to an expert center for biliary drainage.

## Conclusions

To conclude, we propose here a promising tool to predict early death after biliary drainage in MBO that could help physicians plan palliative care and warn patients and their families in high-probability situations. It could be incorporated into a decision-making process to refine patient selection and ultimately avoid futile drainage. However, other factors that can predict mortality probability also should be considered. More data are needed before implementing the score in other situations, such as EUS-guided drainage.

## Notes

Note This article was changed according to the correction on September 25, 2026.
**Correction**
Following publication of this article, the authors identified a unit conversion error in the reporting of the serum creatinine value. The correct serum creatinine threshold is 0.966 mg/dL. This correction was implemented in the online version of the article on 25.09.2026.

## Supplementary Material

Supplementary Material Zusatzmaterial
