# Correction: Which patients with palliative malignant biliary obstruction will benefit most from biliary drainage: Development and validation of a prognostic score

**DOI:** 10.1055/a-2963-7763

**Published:** 2026-10-07

**Authors:** Raphael Olivier, Estelle Antoine, Marie Morvan, Augustin D'Aubigny, Jean Baptiste Nousbaum, Noemie Reboux, Enrique Perez Cuadrado Robles, Lucille Queneherve

**Affiliations:** 1 Hepato-Gastroenterology 36655 CHU PoitiersPoitiersVienneFrance; 2 Hepato-Gastroenterology 26990 CHRU de BrestBrestBretagneFrance; 3 Hepato-Gastroenterologie 55151 Centre Hospitalier de CornouailleQuimperFrance; 4 55647 Hôpital Européen Georges PompidouParisÎle-de-FranceFrance



Following publication of this article, the authors identified a unit conversion error in the reporting of the serum creatinine value. The correct serum creatinine threshold is 0.966 mg/dL.

This correction was implemented in the online version of the article on 25.09.2026.

