# Supplementary material for: Which patients with palliative malignant biliary obstruction will benefit most from biliary drainage: Development and validation of a prognostic score
Source: Endosc Int Open. 2026 Jan 21;14:a27606318. doi: 10.1055/a-2760-6318 (PMC12828974; doi:10.1055/a-2760-6318)

Supplementary Table 1 Univariable and multivariable analyses of factors predictive of 30-day mortality.

		Univariable analysis		Multivariable analysis	
**Characteristic**	**N**	**OR (95% CI)**	***P* value**	**OR (95% CI)**	***P* value**
**Male sex**	262	0.52 (-0.18-1.27)	0.1		
**Age <75**	262	0.47 (-1.20-0.23)	0.2		
**BMI ≥ 26**	249	0.42 (-0.43-1.21)	0.3		
**Hemoglobin <11 g/dL**	260	0.74 (-1.45 to -0.05)	0.04	0.02 (-0.88-0.94)	1
**Platelets ≥322 G/L**	260	0.24 (-0.48-0.95)	0.5		
**Leukocytes ≥ 12 G/L**	260	1.5 (0.76-2.24)	< 0.0001	0.97 (-0.01-1.94)	0.05
**Total bilirubin ≥ 10.8 mg/dl**	258	1.37 (0.47-2.48)	0.006	1.43 (0.33 to -2.72)	0.02
**Urea ≥ 6.5 mmol/L**	258	1.56 (0.84-2.32)	< 0.0001	0.78 (-0.26-1.83)	0.1
**Creatininemia ≥ 5.0 mg/dL**	261	1.79 (1.05-2.54)	< 0.0001	1.54 (0.38 to -2.73)	0.009
**WHO PS**	262				
0-1		Reference			
2		0.72 (-0.13-1.6)	0.1	0.62 (0.40-1.67)	0.2
3-4		1.93 (1.03-2.85)	< 0.0001	1.84 (0.72 to -3.09)	0.003
**Pruritus**	262	0.003 (-0.77-0.72)	1		
**Sepsis**	262	0.025 (-0.99-0.81)	1		
**Comorbidities**					
Cardiopathy	262	0.24 (-0.53-0.97)	0.5		
Chronic renal failure	262	1.45 (0.38-2.45)	0.005	0.29 (-1.20-1.69)	0.7
Chronic respiratory disease	262	0.16 (-1.65-0.97)	0.8		
Obstructive sleep apnea syndrome	262	13.8 (NA-147)	1		
Obesity	262	0.35 (-0.8-1.33)	0.5		
Diabetes	262	0.42 (-0.46-1.21)	0.3		
Stroke	262	0.3 (-1.21-1.49)	0.6		
**Liver metastases**	262	0.94 (0.24-1.65)	0.008	0.91 (-0.08-1.93)	0.07
**Peritoneal carcinomatosis**	262	1.19 (0.35-2)	0.004	0.53 (-0.67-1.72)	0.4
**Other metastases**	262	1.34 (0.63-2.09)	0.0003	1.20 (0.2 to -2.23)	0.02

BMI, body mass index; OR, odds ratio; WHO PS, World Health Organization Performance Status.

Supplementary Fig. 1 AUROC curve for the final predictive model. AUC, area under the curve.

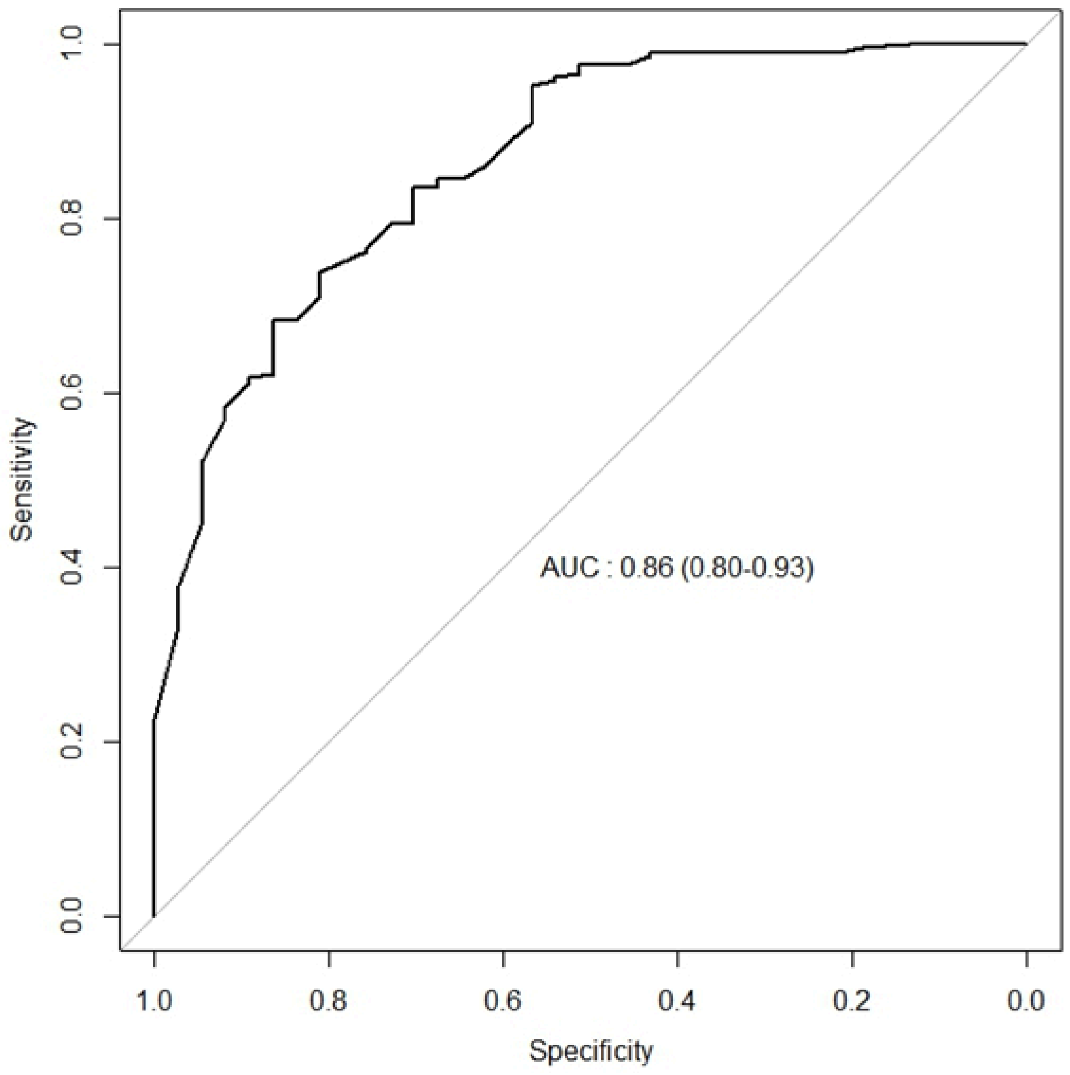

Supplement: Supplementary file 1 — Supplementary Material [file 10-1055-a-2760-6318_27654974.pdf]
